# Histogram analysis of T2 mapping for detecting early involvement of extraocular muscles in patients with thyroid-associated ophthalmopathy

**DOI:** 10.1038/s41598-020-76341-6

**Published:** 2020-11-10

**Authors:** Ping Liu, Lang Chen, Qiu-xia Wang, Ban Luo, Huan-huan Su, Gang Yuan, Gui-hua Jiang, Jing Zhang

**Affiliations:** 1 Department of Medical Imaging, Guangdong Second Provincial General Hospital, Jinan University, Guangzhou, People’s Republic of China; 2 Department of Radiology, The Affiliated Tongji Hospital, Tongji Medical College, Huazhong University of Science and Technology, Wuhan, People’s Republic of China; 3 Department of Ophthalmology, The Affiliated Tongji Hospital, Tongji Medical College, Huazhong University of Science and Technology, Wuhan, Hubei People’s Republic of China; 4 Department of Endocrinology and Metabolism, The Affiliated Tongji Hospital, Tongji Medical College, Huazhong University of Science and Technology, Wuhan, Hubei People’s Republic of China

**Keywords:** Biomarkers, Diseases, Endocrinology

## Abstract

Using histogram analysis of T2 values to detect early involvement of extraocular muscles (EOMs) in patients with thyroid-associated ophthalmopathy (TAO). Five EOMs of each orbit were analyzed for 45 TAO patients and 22 healthy controls (HCs). Patients’ EOMs were grouped into involved or normal-appearing EOMs (NAEOMs). Histogram parameters and signal intensity ratios (SIRs) of EOMs were compared; receiver operating characteristic (ROC) curve analysis was performed to differentiate NAEOMs from EOMs of HCs. 24 patients were reassessed following immunosuppressive treatment. For SIRs, involved muscles showed higher values than those of NAEOMs and HCs (*p* < 0.05); there were no differences between NAEOMs and HCs (*p* = 0.26). Parameters of involved muscles showed no different from those of NAEOMs excluding 25th, 50th percentiles, and standard deviation (SD) (*p* < 0.05). NAEOMs displayed higher values of 90th, 95th percentiles, SD, skewness, inhomogeneity, and entropy than HCs (*p* < 0.05). ROC curve analysis of entropy yielded the best area under the ROC curve (AUC; 0.816) for differentiating NAEOMs and HCs. After treatment, histogram parameters including 5th, 75th, 90th, and 95th percentiles, SD, kurtosis, inhomogeneity, and entropy were reduced in NAEOMs (*p* < 0.05). T2 histogram analysis could detect early involvement of EOMs in TAO prior to detection on conventional orbital MRI.

## Introduction

Thyroid-associated ophthalmopathy (TAO) is an autoimmune disease characterized by orbital tissue inflammation, expansion, fatty infiltration and fibrosis^1^. The current evaluation system is primarily based on the anterior visible part of the orbit and ignores deep structures, such as the extraocular muscle (EOM)^2^. EOM is one of the target organs of immune attack in TAO, its enlargement can produce severe complications^3^. As early as 1979^4^, researchers found that approximately 70% of patients with Graves’ disease, who commonly characterized by subclinical ophthalmopathy, can present with EOM enlargement on computed tomography (CT) or magnetic resonance imaging (MRI). Therefore, the involvement of EOM may begin much earlier than is currently clinically recognized; the ophthalmic signs or symptoms-based evaluation system may be inaccurate, thus, lead to untimely and inappropriate treatment.

Multimodality orbital , for its excellent soft tissue contrast, is viewed as an objective and sensitive tool to assess the EOMs from anatomy to tissue components in TAO^5^. However, most of the MRI methods are either semi-quantitative or based on signal intensity, which are not typically directly proportional to specific tissue properties. Additionally, these techniques mainly quantify disease activity, severity or therapy response; there is minimal attention to detection of the early involvement of EOMs. Politi et al.^2^ and Kilicarslan et al.^6^ suggested that diffusion-weighted imaging (DWI) could detect early asymptomatic involvement of EOMs prior to abnormality on conventional orbital MRI. However, DWI exhibits some inherent limitations in orbital imaging, particularly in terms of susceptibility to artifacts and signal loss^7^.

T2 mapping was considered as a tissue-specific technique can reflect biochemical components within EOMs^8^ and effectively characterize subtle changes from disease process or therapy before they manifest as signal abnormalities^9,10^ However, T2 values are frequently obtained from manually traced regions of interest (ROIs) on a single slice^5,11^, rather than integral muscle, resulting in measurement sampling errors and subjective bias. Recently, application of T2 for TAO have been reported^5,10^, but they also focused on staging and activity and have not yet been used to identify early involvement of EOM, and the information typically provided by mean T2 is rather limited. Volumetric histogram analysis of T2, measure the variations and frequencies of T2 values within whole tissues, can provide comprehensive and informative T2 parameters, is expected to potentially detect early EOM involvement even when there are no changes on conventional images.

Based on previous reports and on our own clinical observations, we developed the hypothesis that all EOMs of TAO patients may experience immune damage during early disease stage. Therefore, we aimed to detect this early involvement using volumetric T2 histogram analysis.

## Results

In this study, 383/450 EOMs of untreated TAO patients were designated as the “involved muscle group” (n = 383) and 67/450 were designated as the “normal-appearing muscle group (NAEOMs)” (n = 67) by assessment of muscle involvement on conventional T2WI images. In addition, 220 EOMs of healthy controls (HCs) were included. In total, 191/240 involved EOMs and 49/240 NAEOMs of 24 patients who received treatment were also compared following therapy.

### Interobserver agreement

Overall, the inter-observer agreement between the two readers was good to excellent for SIRs and volumetric T2RT histogram parameters (Table 1; intraclass correlation coefficients ranged from 0.785 to 0.998). Therefore, the T2RT histogram analysis was performed based on the results of the first neuroradiologist.

**Table 1:** The interobserver agreement between two radiologists for histogram parameters and SIR.

	Healthy Controls		TAO patient before therapy		TAO patient after therapy	
	ICC	95% CI	ICC	95% CI	ICC	95% CI
SD	0.876	0.825–0.912	0.995	0.993–0.996	0.955	0.940–0.971
Skewness	0.962	0.950–0.972	0.956	0.941–0.967	0.908	0.863–0.908
Kurtosis	0.913	0.885–0.935	0.961	0.948–0.971	0.902	0.853–0.931
Entropy	0.953	0.938–0.965	0.892	0.857–0.918	0.960	0.941–0.973
Inhomogeneity	0.822	0.718–0.888	0.967	0.955–0.975	0.922	0.884–0.961
T2_5%_ (ms)	0.852	0.765–0.906	0.988	0.984–0.991	0.995	0.993–0.997
T2_10%_ (ms)	0.906	0.843–0.943	0.981	0.974–0.986	0.894	0.831–0.933
T2_25%_ (ms)	0.985	0.980–0.989	0.957	0.943–0.968	0.994	0.993–0.997
T2_50%_ (ms)	0.924	0.899–0.943	0.935	0.913–0.951	0.879	0.809–0.924
T2_mean_ (ms)	0.894	0.831–0.933	0.998	0.997–0.998	0.914	0.872–0.942
T2_75%_ (ms)	0.917	0.890–0.937	0.954	0.939–0.966	0.890	0.826–0.930
T2_90%_ (ms)	0.919	0.893–0.939	0.954	0.939–0.966	0.889	0.836–0.926
T2_95%_ (ms)	0.920	0.895–0.940	0.930	0.907–0.948	0.876	0.825–0.912
SIR	0.785	0.705–0.857	0.810	0.690–0.880	-	-

The unit of T2 is ms.

*TAO *thyroid-associated ophthalmopathy, *ICC *interclass correlation coefficient, *CI *confidence intervals, *SIR *signal intensity ratio.

### SIR for patients and HCs

Average SIRs were 1.08 ± 0.2 in the involved muscle group, 1 ± 0.14 in the NAEOM group, and 0.97 ± 0.19 in the HCs. Statistical differences were observed between involved and HCs (*p* < 0.001), and between involved and NAEOMs (*p* = 0.04). There was no statistical difference between NAEOMs and HCs (*p* = 0.21) (Table 2).

**Table 2:** Comparison of T2RT histogram parameters and SIRs between untreated TAO patients and control subjects.

Parameters	Healthy controls	Normal-appearing	Involved	*P*	*P1*	*P2*	*P3*
SIR	0.97 ± 0.19	1 ± 0.14	1.08 ± 0.2	< 0.001	0.04	< 0.001	0.21
T2_5%_ (ms)	54.39 ± 8.97	53.82 ± 6.25	56.05 ± 7.04	0.02	0.06	0.17	0.85
T2_10%_ (ms)	56.91 ± 7.9	57.72 ± 5.52	59.72 ± 6.68	0.1	0.5	< 0.001	1
T2_25%_ (ms)	62.3 ± 8.02	63.32 ± 5.41	65.82 ± 7.02	< 0.001	0.03	< 0.001	0.97
T2_50%_ (ms)	68.29 ± 9.18	69.73 ± 5.94	72.77 ± 8.01	< 0.001	0.02	< 0.001	0.51
T2_mean_ (ms)	69.46 ± 9.11	71.44 ± 5.68	74.24 ± 7.98	< 0.001	0.14	< 0.001	0.07
T2_75%_ (ms)	74.94 ± 10.52	77.07 ± 6.48	80.15 ± 9	< 0.001	0.1	< 0.001	0.15
T2_90%_ (ms)	81.07 ± 11.36	84.53 ± 7.6	87.54 ± 10.26	< 0.001	0.25	< 0.001	0.03
T2_95%_ (ms)	86.4 ± 12.21	90.39 ± 7.7	92.36 ± 11.4	< 0.001	0.99	< 0.001	0.02
**SD**	**8.86 ± 3.22**	**9.77 ± 2.14**	**11.17 ± 3.06**	**< 0.001**	**0.008**	**< 0.001**	**0.03**
Skewness	0.17 ± 0.43	0.32 ± 0.43	0.40 ± 0.39	0.002	0.97	0.001	0.03
Kurtosis	0.13 ± 0.74	0.35 ± 0.65	0.31 ± 0.79	0.06	0.59	0.003	0.11
Entropy	3.31 ± 0.14	3.38 ± 0.38	3.45 ± 0.31	< 0.001	0.33	< 0.001	0.04
Inhomogeneity	0.14 ± 0.03	0.15 ± 0.02	0.16 ± 0.03	**< 0.001**	**0.008**	< 0**.001**	**0.01**

The unit of T2 is ms.

*P* value represents the statistical difference among the three groups; *P1* represents the comparison *P* value between involved and normal-appearing group. *P2* represents the comparison *P* value between involved and healthy controls; *P3* represents the comparison *P* value between normal-appearing and healthy controls.

*P* < 0.05 is considered statistically significant. The bold vlaues represent the results with significant statistical difference.

### Comparison of T2 histogram parameters

**Figure 1:**
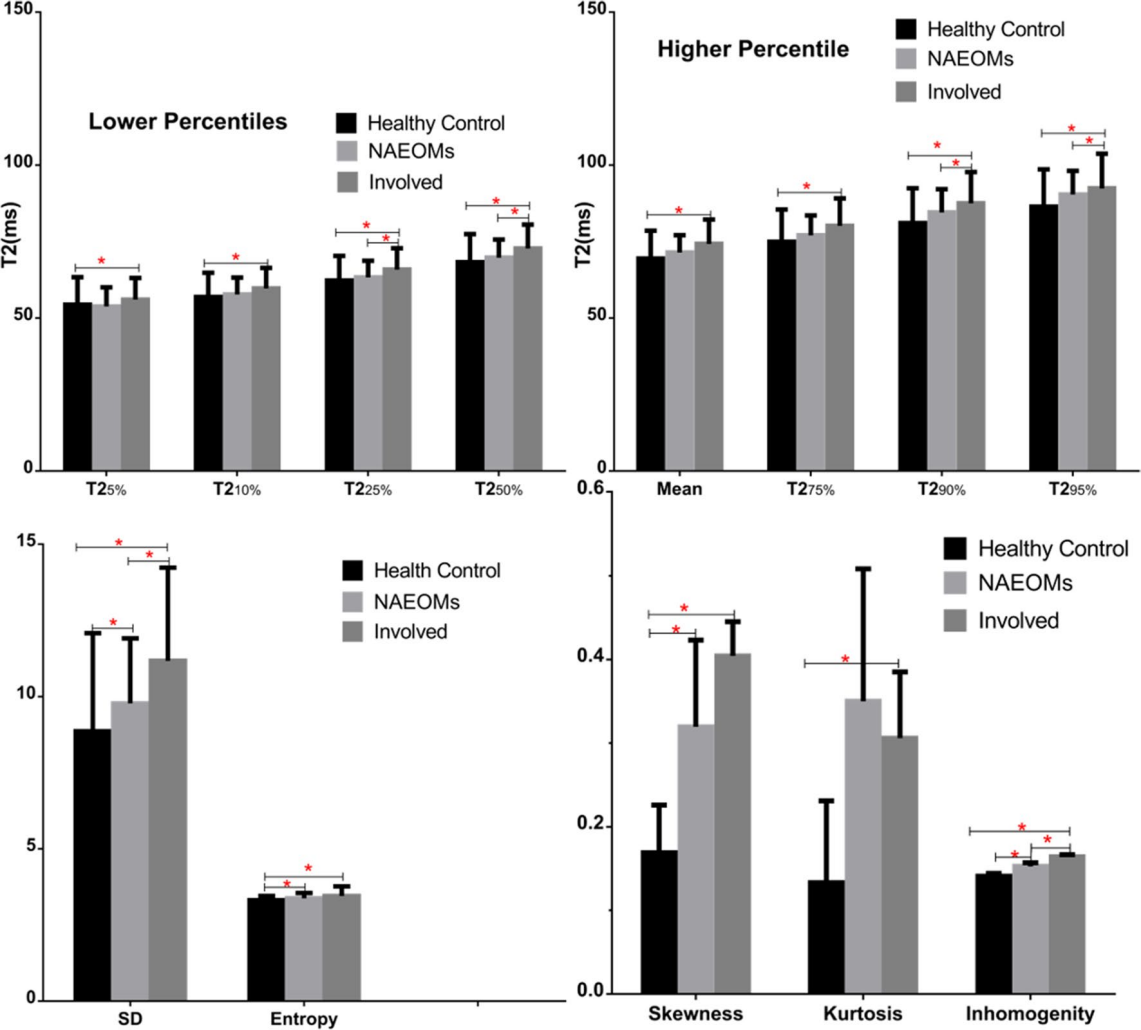
Bar chart of histogram-based texture features of extraocular muscles for the three groups (involved, normal-appearing extraocular muscles, and healthy control subjects). Notes: red asterisk ( ) and red line segment were respectively stand for the significant differences between groups.

**Figure 2:**
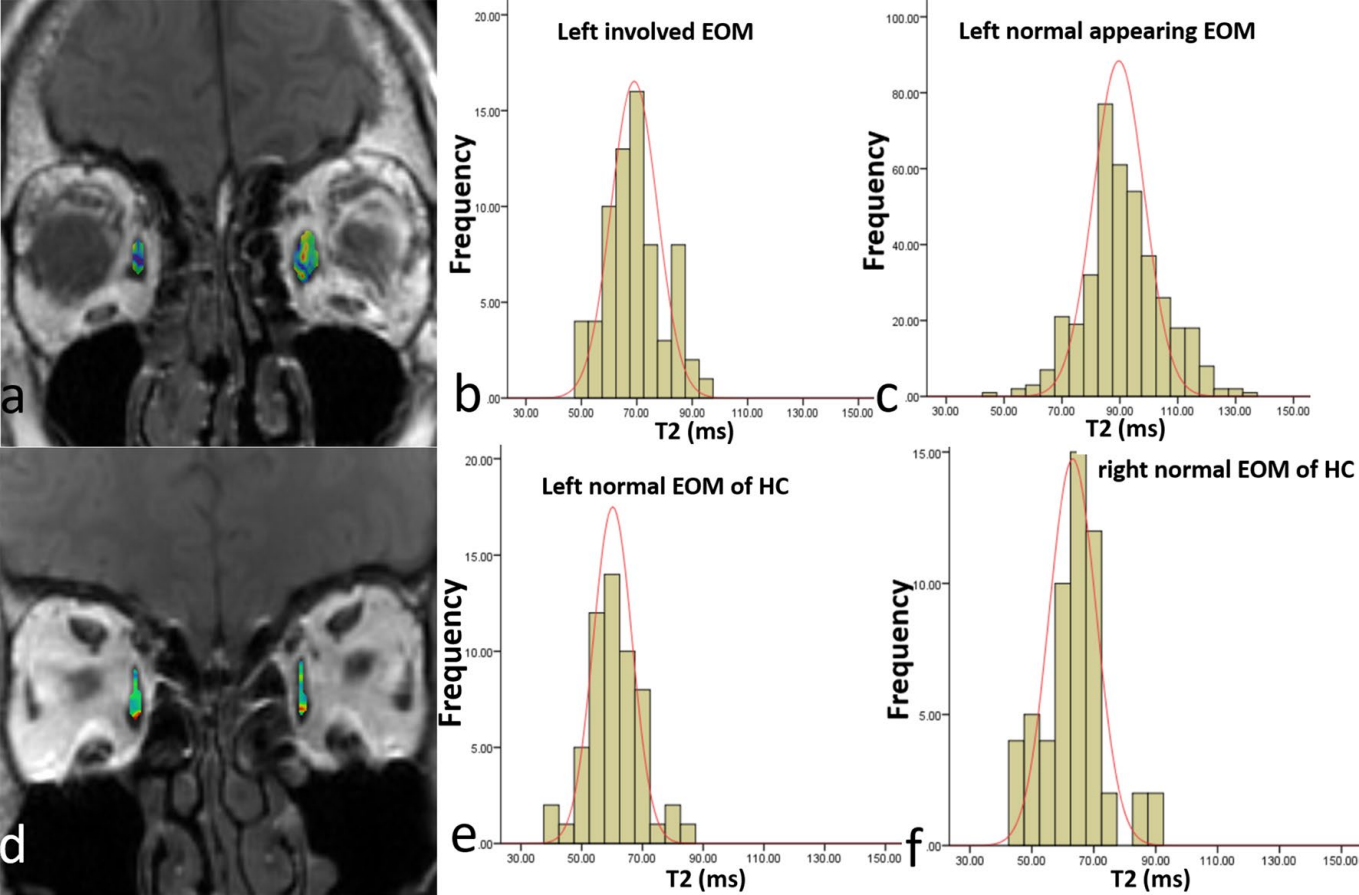
T2 value histogram analysis of a 63-year old TAO patient (**a**–**c**) and a sex and age-matched healthy control subject (**d**–**f**). The top figure (**a**–**c**) were from a 63-year old TAO patient; the bottom figure (**d**–**f**) were from a sex and age-matched healthy control subject. (**a**) The colorful ROI on the left represents the involved left medial rectus (MR), and the colorful ROI on the right stands for the normal-appearing right MR. (**b**) The histogram T2 parameters of involved left MR. (**c**) The histogram T2 parameters of right normal appearing MR. (**d**) The colorful ROI represents the left and right MR of a sex and age-matched healthy control subjects. (**e**,**f**) The histogram parameters of left MR and right MR in the healthy control. Note: due to the chemical shift phenomena between muscle and fat, the signal of the marginal muscles were not the true signal of muscle, therefore, we excluded this part out of the ROI to ensure the measurement accuracy.

As summarized in Table 2 and Fig. 1, all histogram scalars of involved EOMs, excluding the 5th percentile T2, were higher than those of their HCs counterparts. Discrepancies were evident in higher percentiles (90th and 95th) of T2; there were also differences in SD, skewness, entropy, and inhomogeneity of NAEOMs, relative to those of HCs. Excluding 25th and 50th percentile T2RTs, as well as the SD and inhomogeneity, the remaining histogram parameters of NAEOMs did not differ from those of the involved muscles. A representative case is shown in Fig. 2.

### Diagnostic performance of imaging parameters in discerning NAEOMs of TAO from EOMs of HCs

**Figure 3:**
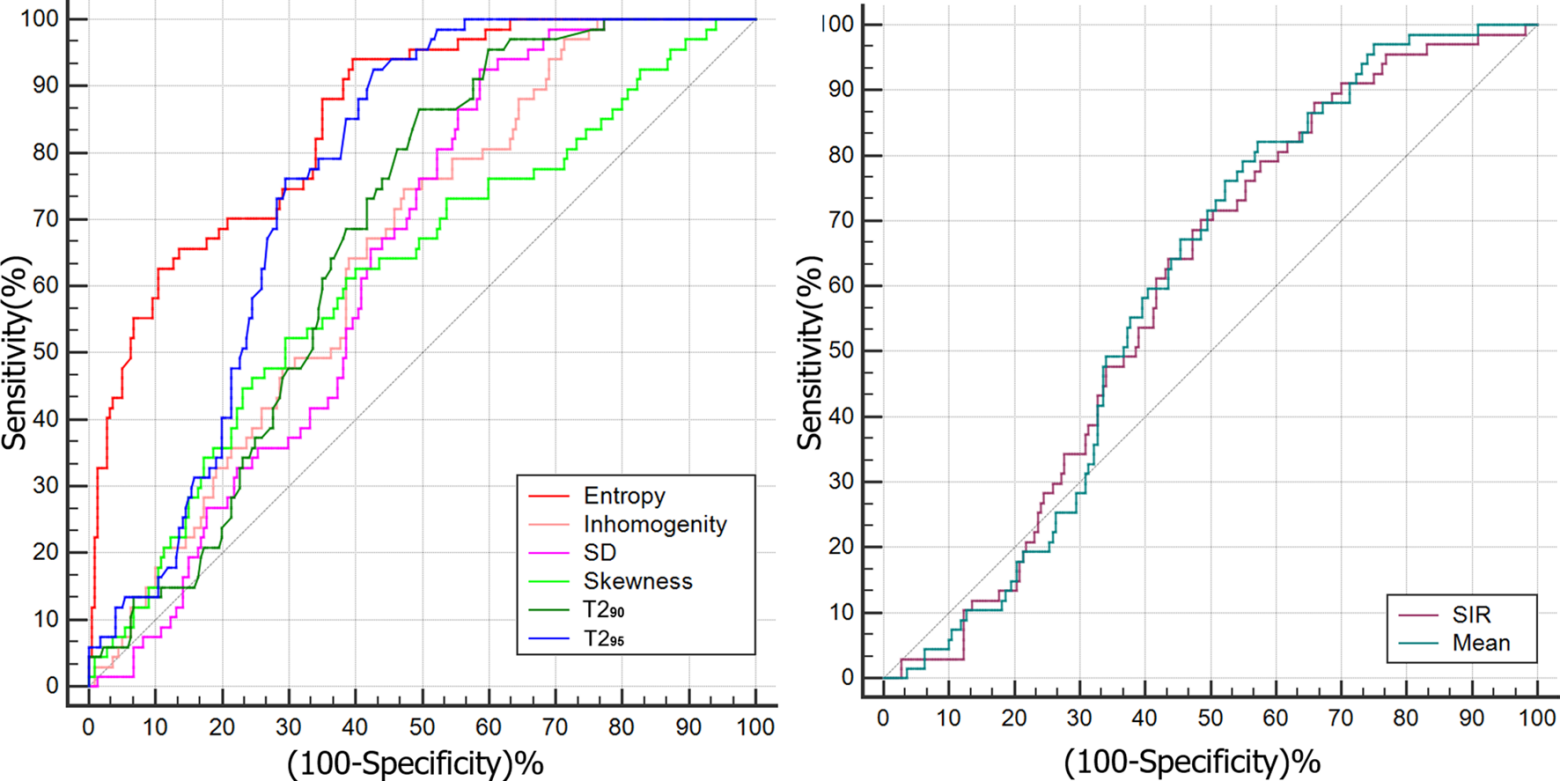
ROC analysis of every significant T2RT histogram parameters, T2RT_mean_ and SIRs. (**a**) The ROC analysis of every significant T2 histogram parameters, the entropy has the highest diagnostic efficiency and its AUC is 0.816. (**b**) The ROC analysis of SIRs from conventional MRI and the mean T2 value (usually used on previous research).

ROC curve analyses of the SIR, mean, 90th, 95th percentiles, SD, skewness, entropy, and inhomogeneity between NA EOMs and EOMs from HCs were performed. As shown in Table 3, entropy and 95th percentiles generated the highest AUC (AUC = 0.816, 0.724; respectively) in their categories than other histogram parameters and SIRs. The entropy had the detective performance with the estimated cutoff of 3.292 (AUC, 0.816; 95% confidence interval: 0.767, 0.859) (Fig. 3), and corresponding sensitivity and specificity were 94% and 59.1%, respectively (Table 3).

**Table 3:** Diagnostic performance of histogram parameters of the T2RT value in discerning normal-appearing EOMs from healthy EOMs.

Parameter	Cutoff value	AUC	P	Sensitivity (%)	Specificity (%)
SIR	> 0.856	0.531 (0.472–0.590)	0.381	80.6	35
Mean (ms)	> 66.6	0.599 (0.540–0.656)	0.004	82.1	42.7
Skewness	> 0.392	0.621 (0.562–0.677)	0.002	52.2	70.5
SD	> 7.308	0.639 (0.581–0.695)	< 0.001	92.5	41.4
Inhomogeneity	> 0.143	0.653 (0.595–0.708)	< 0.001	74.6	52.7
T2_90%_ (ms)	> 78.6	0.667 (0.609–0.721)	< 0.001	86.6	48.6
T2_95%_ (ms)	> 85.8	**0.724 (0.668–0.774)**	< 0.001	93.5	49.5
Entropy	> 3.292	**0.816 (0.767–0.859)**	< 0.001	91.1	57.3

Numbers in parentheses are 95% confidence intervals.

The unit of T2 is ms.

The bold vlaues represent the results with significant statistical difference.

### Comparison of T2 histogram parameters for patients who accepted therapy

All histogram parameters of involved muscles significantly declined after therapy, with the exception of skewness (*P* = 0.16). Heterogeneous metrics, in addition to the 5th and higher (75th, 90th, 95th) percentiles of T2 of NAEOMs showed marked reductions after therapy (Table 4).

**Table 4:** Comparison of T2RT histogram parameters for TAO patients before and after therapy.

Parameters	Involved muscles			Normal appearing muscles		
	Pre	Post	*P*	Pre	Post	*P*
Mean (ms)	74.19 ± 7.57	70.12 ± 7.4	< 0.001	70.79 ± 5.11	69.91 ± 5.29	0.23
T2_5%_ (ms)	55.37 ± 6.58	54.33 ± 7.26	0.04	53.13 ± 6.07	55.34 ± 5.99	0.04
T2_10%_ (ms)	59.08 ± 6.43	57.33 ± 6.16	0.001	57.02 ± 5.5	57.96 ± 5.58	0.32
T2_25%_ (ms)	65.41 ± 6.7	62.76 ± 7.23	< 0.001	62.73 ± 4.69	63.11 ± 4.94	0.61
T2_50%_ (ms)	72.8 ± 7.5	70.13 ± 6.94	< 0.001	68.98 ± 4.86	68.4 ± 5.56	0.43
T2_75%_ (ms)	82.16 ± 9.04	68.86 ± 7.57	< 0.001	76.35 ± 5.69	74.79 ± 6.3	0.03
T2_90%_ (ms)	88.06 ± 10.37	81.68 ± 9.52	< 0.001	84.26 ± 8.31	81.3 ± 7.29	0.01
T2_95%_ (ms)	92.88 ± 11.53	85.98 ± 10.66	< 0.001	89.78 ± 12	85.39 ± 7.94	0.02
SD	11.34 ± 3.07	9.22 ± 2.72	< 0.001	10.48 ± 2.7	8.66 ± 2.35	< 0.001
Skewness	0.25 ± 0.4	0.18 ± 0.39	0.16	0.38 ± 0.45	0.27 ± 0.39	0.12
Kurtosis	0.30 ± 1	− 0.01 ± 0.55	0.001	0.45 ± 0.81	− 0.06 ± 0.63	< 0.001
Entropy	3.54 ± 0.28	3.14 ± 0.38	< 0.001	3.37 ± 0.27	2.97 ± 0.36	< 0.001
Inhomogeneity	0.15 ± 0.03	0.13 ± 0.04	< 0.001	0.15 ± 0.37	0.13 ± 0.33	< 0.001

‘Pre’ represents the patients before therapy; ‘post’ represents the patients after therapy.

Statistical significance is indicated by *P* values less than 0.05.

The unit of T2 is ms.

## Discussion

This study exploited multiple T2 measures using histogram analysis techniques on a pixel-by-pixel basis to investigate subtle changes of EOMs in patients with TAO. This novel method beyond conventional mean T2 measures to evaluate the percentiles and heterogeneity within and among muscles with distinct statuses. We found that: (1) elevated percentiles and increased heterogeneous distribution of T2 can occur in all EOMs of TAO patients, including involved or NAEOMs on conventional MRI; (2) the NAEOMs exhibited similar changes in percentiles and non-uniform T2 distribution in comparison to involved muscles; (3) NAEOMs showed much higher values in 90th and 95th percentiles T2 than those of HCs.

We found that the involved EOMs exhibited overt increase in point-specific parameters (excluding the 5th percentile) and more inhomogeneous distribution of the T2 than EOMs of HCs. Histogram features provide novel quantifications of the intrinsic distributions of different tissues, such as percentiles providing a greater range of T2 values to reveal complex tissues; these have been widely used to quantify the T2 distribution in muscles such as myocardium^12,13^ and pelvic limb muscles^14^. Ishu et al.^9^, demonstrated that the lower leg muscles of boys with Duchenne muscular dystrophy exhibited elevated percentiles and heterogeneities on T2-mapping histogram analysis. Inflammation, fatty degeneration and fibrosis were main pathological changes of EOMs in patients with TAO, there is no absolute boundary between the fatty infiltration and edematous, and both of them can lead to increased T2 values^15^. Heterogeneous expression at the cellular and molecular levels, including fibroblast phenotypes, cytokine profiles, and disparate T cell subsets^3,16^, may produce the asynchronous and non-uniform pathological changes in each EOM within the same orbit, thus the microstructural component distribution within each EOM may be uneven. The varying deposition of macromolecules (e.g., collagen), interstitial edema, and fibrous scar tissue and fatty infiltration within the involved muscles can result in greater distribution of T2 values^17,18^. Factually, uneven distribution of pathological components in EOMs in patients with TAO has been reported by Yokoyama et al.^19^ and it was observed to have correlation with the therapeutic response to corticoid treatment. However, the uniformity in Yokoyama’s report was based on visual observation of T2 signal intensity of EOMs on conventional MRI; notably, T2 SI is variable and does not directly reflect tissue properties, such as density or molecular composition.

Interestingly, the NAEOMs exhibited larger values in both inhomogeneity metrics and higher percentiles (90th and 95th) of T2 values, relative to those of HCs. However, no differences were observed in the SIR, mean, and lower percentiles of T2. Our findings regarding the early involvement are similar to, but more obvious than, those reported by Kilicarslan et al.^6^, who observed changes in diffusion values but without significant differences. We speculate the changes in NAEOMs are from the following factors: (1) previous T2 studies on EOMs in TAO primarily focused on altered T2, averaged over one or several image slices^11,20^, which may harbor or mask small pathological changes. These subtle pathologies could be diffusely or focally distributed throughout the EOM, such that they could not be captured by conventional MRI. Histogram-derived percentile parameters, in which the T2 values were arranged from small to large, may enable distinction of subtle differences. (2) Lower percentiles of T2 represent authentically normal muscle area, while the mean and higher percentiles (75th, 90th, and 95th) of T2 may represent abnormal voxels. In NAEOMs, large amounts of normal voxels masked tiny amounts of abnormality; consequently, only very high percentiles of T2 reflecting extremely abnormal voxels could detect this subtle modification. (3) Secondary to long-standing systemic immunoreactions, NAEOMs also experience lymphocyte infiltration and accumulation of granular material. These microstructural changes precede functional, morphological, and signal changes, which may be a hallmark of early muscle involvement and are difficult to discern by conventional means.

Notably, overtly involved muscles displayed higher 25th and 50th percentiles, compared to those of NAEOMs. This is potentially because both 25th and 50th percentiles were located in borderline areas between minimal abnormality and disease. Most of the distribution shape-related scalars of NAEOMs (with the exception of SD and inhomogeneity) displayed no obvious differences from those of involved muscles. These results further supported the immunologic injury in NAEOMs.

In our present findings, the entropy had the best diagnostic performance among the T2 histogram parameters and SIRs. Both the SIRs on conventional MRI and the usually used T2_mean_ value had lower AUC when compared to the higher T2 percentiles and entropy. It demonstrated that conventional MRI features and commonly used T2 may have some limitations in detecting the early changes of EOMs in TAO. The entropy refers to irregularity of gray-level distribution, higher entropy reflects greater complexity of specific tissue^21^ and indicates the diversity tissue component. This finding suggest that the quantification of macromolecules can be used as a direct imaging marker of early involvement.

In this study, in patients who displayed clinical amelioration, apart histogram parameters for involved EOMs changed. The 5th and higher (75th, 90th, 95th) percentiles of T2 and all uniformity-related parameters of NAEOMs decreased markedly after therapy. Therapy-induced modifications of NAEOMs indirectly demonstrated that they exhibited some pathology.

Our results are consistent with the immunological nature of TAO: when immune activity is triggered, all EOMs experience varying degrees of immune reactions. EOM damage begins much early prior to the onset of overt clinical signs and symptoms, as well as before detection on routine orbital MRI; thus, comprehensive and meticulous evaluations should be performed to detect such changes. This rationale explains the phenomenon we frequently encountered in clinical practice: many patients who initially manifested with mild involvement in some EOMs, which quickly progressed to severe ophthalmopathy and involved all EOMs. It can also partially explain why local therapy is less effective than systemic intravenous injection, even in TAO patients with isolated muscle involvement. Thus, our results may promote recognition of the traditional definition of “non-involvement” based on clinical assessment, gross morphology or signal intensity on conventional MRI. The histogram parameters of T2 may offer an data-based evidence to facilitate improved clinical treatment of TAO.

### Limitations

This study had some limitations. First, it did not include a “gold standard,” such as histological or immunohistological analyses, which could have verified the microscopic alterations behind the image analysis. Notably, this verification may be conducted in appropriate animal models. Second, the neuroradiologists were aware of the subjects’ conditions when identifying muscle involvement on conventional T2WI images; they assumed that the EOMs of HCs were normal and did not assess their involvement. Third, the T2 mapping technique were performed without fat suppression, and we cannot really distinguish the altered value were from fat or edemas, but both of them were pathology changes in TAO. Fourth, the T2 values calculated in this research still failed to overcome the inherent drawbacks that the contamination from stimulated and indirect echoes, which thus give rise to deviated and inaccurate T2 values. Finally, the sample size of NAEOMs was limited, and patients were not classified into subgroup according to varying activity, which may have reduced the statistical power of the study.

## Conclusion

The combination of histogram analysis and T2-mapping imaging can detect early pathology in EOMs without obvious involvement on conventional orbital MRI. This multiparametric approach may provide a breakthrough for exploring early injuries in EOMs for patients with TAO. Early intervention may be started in patients with highly abnormal histogram parameters regardless of the CAS score and when there is no evidence of EOM involvement on conventional imaging. This information may aid in the management of TAO.

## Materials and methods

### Study patients

This retrospective study was approved by the institutional review board of Tongji Hospital, Tongji Medical College, Huazhong University of Science and Technology. Written informed consent was obtained from all patients for MRI scanning, but the written informed consent of the data usage for this research was waived as the MRI protocol is conventional in our hospital and this is a retrospective study.

Between May 2017 and Apr 2018, TAO patients and healthy controls (HCs) were recruited. An endocrinologist and an ophthalmologist jointly diagnosed the TAO according to the consensus of the European Group on Graves' Orbitopathy^22^. Patients were enrolled if they met the following criteria: (1) initially diagnosed with active moderate-severe TAO; (2) showed no other accompanying orbital disease; (3) had no prior orbital therapy or surgery; (4) successfully completed MRI examinations.

Finally, 45 active moderate-severe TAO patients (25 women, 20 men; mean age, 48.1 ± 11 years; range, 25–65 years) met the inclusion criteria. In addition, 22 HCs (12 women, 10 men; mean age, 45.3 ± 12 years; range, 25–64 years) were included; they were euthyroid healthy volunteers without any ocular symptoms or signs of exophthalmos (diplopia, itching, or reduced eye mobility).

24 patients who completed therapy and exhibited a favorable therapeutic response were further assessed. And all of them were received the standard therapy regimen which were recommended by 2016 EUGOGO guidelines: intravenous methylprednisolone (Pfizer Manufacturing Belgium NV) 0.5 g per week for 6 weeks, followed by 0.25 g per week for 6 weeks. The total therapy course lasted for 12 weeks.

### Orbital MRI

Orbital MRI was performed 1 week before the initial treatment for patients. Patients who underwent therapy were re-scanned within 2 weeks after the completion of therapy. All MRI scans were performed on a 3T MRI scanner (Discovery 750, GE Healthcare, Milwaukee, WI, USA) with a 32-channel head coil. Subjects were asked to keep their eyes closed during scanning, to facilitate the subjects to keep the eye closed, some auxiliary does not interfere with magnetic fields were covered on their eyes.

The MRI protocol was as follows: (1) axial T2WI (retention time [TR]/echo time [TE], 2800/68 ms; slice thickness, 3 mm; intersection gap, 1 mm); (2) coronal T2-IDEAL (TR/TE, 2200/68 ms; slice thickness, 3 mm; intersection gap, 0.6 mm). (3) coronal T2-mapping with eight consecutive echoes (TE, 9.9–79.2 ms; ΔTE, 9.9 ms) and the following parameters: TR, 2000 ms; section thickness of 3 mm, gap of 0.6 mm; FOV, 180 × 180 mm; matrix, 256 × 256; and bandwidth, 15.63 kHz/pix. Acquisition time for T2-mapping was approximately 6 min 24 s.

The axial T2WI images were traditional orbital images to exclude other obvious ocular disease. The T2-IDEAL technique was utilized to observe morphological changes and assess SIRs. The coronal T2-mapping sequence was employed to assess T2 values.

### Image analyses

The following image analyses were performed by two experienced neuroradiologists with more than 7 years of experience. The neuroradiologists were blinded to the clinical data and subjects’ conditions (patients or HCs), although the images may have suggested some characteristics of the subjects.

(1) Signal intensity measurements

**Figure 4:**
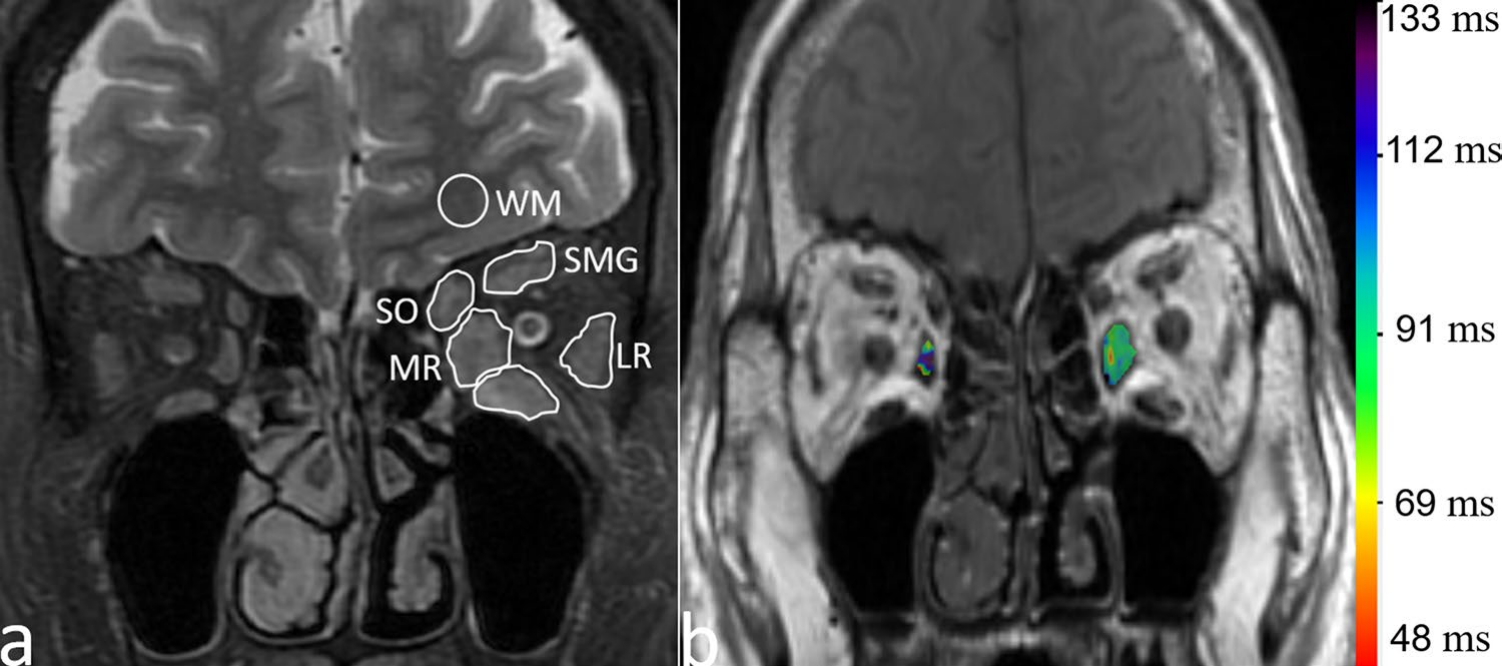
Sketch option of the generation of ratio of the signal intensity and histogram analysis. (**a**) Measurement of signal intensities of extraocular muscles using iterative decomposition of water and fat with echo asymmetric and least-squares estimation (IDEAL) image. (**b**) T2 overlay map of the left medial rectus, the rainbow bar represents the range of T2 values in this specific volume of interest (VOI). Notes: the signal intensities of each muscle and the brain were measured as shown in the left orbit and brain. To standardize the signal intensity of each muscle, the SIR in each muscle to that in white matter of the same slice was calculated. *IR* inferior rectus, *IR* lateral rectus, *MR* medial rectus, *SO* superior oblique, *SMG* superior muscle group, *WM* white matter.

SIRs of all subjects were measured using the “average” method, as described in previous studies^23,24^, on a GE AW 4.6 workstation (GE Healthcare). The neuroradiologists manually outlined the ROIs of five EOMs (superior, inferior, medial, lateral rectus, and superior oblique muscles) for each orbit. They delineated the muscles on the slice with the most prominent signal intensity alterations on water images of the coronal T2-IDEAL sequence. Because separation of the superior rectus and levator palpebrae superioris is difficult, these muscles were sketched together as the superior muscle group (Fig. 4a).

To standardize the signal intensity of the EOMs, the white matter was used as the reference standard tissue, due to its robust reproducibility. Finally, the mean SIR was calculated.

(2) Volumetric T2 histogram analysis

MRI data for the T2 maps were digitally transferred to a personal computer and processed with custom-developed software (Fire Voxel, New York University, New York City, NY, USA). Post-processing was conducted using the monoexponential T2-mapping fitting model.

All ROIs of EOMs were traced by the two neuroradiologists. The ROIs were manually drawn on all contiguous slices (selecting a TE value that best depicted the EOMs) along the edge of the EOMs to encompass the whole muscle. The neuroradiologists were careful to mitigate partial volume effects from orbital fat and air-filling in the paranasal sinuses (Fig. 4b).

Based on histogram analysis of the ROIs, T2 histograms and a variety of parameters were generated through combined analysis by Fire Voxel and SPSS software (version 24, IBM Corp., Armonk, NY, USA). These parameters were divided into two categories^25^, including (1) point-specific parameters, which directly mirrored the T2 values: mean (representing average values within a specific ROI) and other cumulative T2 histograms: lower percentiles (5th, 10th, 25th, 50th) , higher percentiles (75th, 90th, 95th), where the nth percentile was the point at which n percent of voxel values were situated on the left area of the histogram^26,27^; and (2) histogram shape-related parameters, which indirectly reflected the heterogeneous distribution of T2: (a) standard deviation (SD, measuring degree of dispersion from the mean value); (b) skewness (reflecting asymmetry of the histogram distributions); (c) kurtosis (representing the peaked nature of the histogram); (d) entropy (describing irregularity or complexity of the distribution of a parameter of interest); and (e) inhomogeneity (quantifying intralesional heterogeneity).

### EOM involvement evaluation

EOMs were divided into involved or normal-appearing in a binary manner. Involvement was visually evaluated as the presence of swelling and brightness signal intensity (with reference to the temporalis) on T2-IDEAL imaging. EOMs without these manifestations were designated as normal-appearing extraocular muscles (NAEOMs). The two neuroradiologists determined muscle involvement for TAO patients, but not for HCs. Disagreements regarding classification were resolved by a third neuroradiologist with 15 years of experience. The SIR and T2 parameters were classified based on the involvement evaluation.

### Statistical analysis

Data are expressed as the mean ± SD. A significance level of P < 0.05 was considered statistically significant. All statistical analyses were performed using the SPSS statistical software package.

Interobserver agreement for histogram parameters and SIRs was assessed using the intraclass correlation coefficient. The Kolmogorov–Smirnov test was used to assess the normality of continuous variables with regard to the SIRs and histogram parameters. Based on the results of the Kolmogorov–Smirnov test, comparisons among the three groups were performed using the ANOVA or independent-samples Kruskal–Wallis test. Receiver operating characteristic (ROC) curves were constructed to evaluate the diagnostic performance of the SIRs and T2RT histogram parameters to differentiate the really normal EOMs and the early involved EOMs (that is NAEOMs). Paired *t*-tests or paired Wilcoxon signed-rank tests were conducted to investigate changes in histogram parameters and SIRs of EOMs in patients who achieved good therapeutic responses.

### Compliance with ethical standards

This study was approved by the Institutional Review Board (IRB) of Tongji Hospital, Tongji Medical College, Huazhong University of Science and Technology, and we pledged to abide by the declaration of Helsinki (2000 EDITION) in accordance with the relevant medical research rules of China in the study. Written informed consent was obtained from all patients for MRI scanning, but the written informed consent of the data usage for this research was waived as the MRI protocol is conventional in our hospital and this is a retrospective study. All patient-sensitive information was kept with confidentiality and used only for the purpose of the study.

### Ethical approval

All procedures performed in the studies involving human participants were in accordance with the ethical standards of our institutional review board and with the 1964 Helsinki Declaration and its later amendments or comparable ethical standards.

### Informed consent

Written informed was accepted from all patient.
